# 中国医师协会胸外科医师分会创建、成立与发展十年回顾

**DOI:** 10.3779/j.issn.1009-3419.2018.03.03

**Published:** 2018-03-20

**Authors:** 天佑 王

**Affiliations:** 100050 北京，首都医科大学附属北京友谊医院胸外科 Department of Toracic Surgery, Beijing Friendship Hospital, Capital Medical University, Beijing 100050, China

**Keywords:** 医师, 学会, 胸外科, Physician, Society, Department of thoracic surgery

中国医师协会胸外科医师分会于2007年1月成立，至今已走过了十年的历程。中国医师协会胸外科医师分会是依法注册、自愿组成的中国普胸外科医师唯一的全国性、行业性协会。它的成立和发展，在中国普胸外科发展的历史进程中、在中国普胸外科医师职业与行业发展中都具有十分重要的历史和现实意义。

中国胸部外科起步于20世纪30年代，包括普胸外科和心血管外科。1985年，中华医学会胸心血管外科学分会成立，促进了中国胸心血管外科的飞速发展，成为中国胸部外科发展史上的里程碑。由于体外循环和心脏直视手术的迅速发展，胸部外科逐步分成了普胸外科和心脏大血管外科两个专业。1997年，第一届全国普胸外科学术大会在北京召开，成为普胸外科全国性独立专科交流和进一步成立普胸外科学术与行业组织的尝试。

2002年，原卫生部委托中国医师协会制定"全国专科医师培训计划、标准和基地遴选"等试行细则，胸外科方面邀请王天佑教授和甄文俊教授负责起草和制定，历时两年顺利完成了此项工作。在此过程中，时任中国医师协会会员部主任谢启麟教授、时任中国医师协会秘书长陆军女士与时任中华医学会胸心血管外科学分会秘书长的王天佑教授，多次协商和酝酿关于组建成立胸外科医师分会的事宜。

经过4年的精心准备，由当时北京医学会胸心血管外科学分会的王天佑教授、支修益教授、张志庸教授、甄文俊教授等专家和天津的张逊教授、上海的高文教授和周允中教授、广东的戎铁华教授、南京的叶玉坤教授等九人组成筹备组，讨论制定了分会的章程、会标、组织办法，并确定了分会的性质是中国胸外科医师的、带有学术性的全国性行业组织，分会的宗旨是团结、自律、维权、发展，分会办公室设在首都医科大学附属北京友谊医院。

在中国医师协会的领导和全国胸外科医师的支持下，2007年1月19日，在北京华润饭店召开大会，全国普胸外科医师专属的、带有学术性的行业组织——中国医师协会胸外科医师分会正式成立。为中国普胸外科医师的自身建设和普胸外科事业发展开辟了广阔的前景。

中国医师协会胸外科医师分会成立十年来，会员人数由最初的近400名发展为近4, 000名，占全国胸外科医师的2/3，规模空前壮大，不断团结奋进，成就令人瞩目。在中国医师协会总会的领导下，在普胸外科学术交流、青年医师规范化培训机制的建立和改革、青年医师微创胸外科技术培训、会员发展等方面做了大量工作。分会成立了微创外科、食管、肺、纵隔及胸壁、创伤、快速康复外科等6个专家委员会和1个手汗症专家组，以及胸外科术语标准化委员会、全国医师定期考核胸外科专业编委会2个专门委员会，制定了各种普胸外科疾病诊疗技术的共识和规范，积极鼓励创新与推广，对于普胸外科的学科和学术发展起到了巨大的推动作用。分会十分重视对全国普胸外科医师的人文教育，设立多种青年医师奖励，设立"中国胸外科杰出贡献奖"、多次表彰胸外科老前辈老专家医德高尚、刻苦钻研、艰苦奋斗、勇于创新的精神和为中国胸外科事业奉献一生的伟大业绩，为全国青年胸外科医师树立了光辉榜样！

分会成立之后，自2007年-2016年连续十年，先后在北京、江苏南京、天津、广东东莞、上海、重庆、四川成都、北京、浙江杭州、河南郑州等地，举办了十届年会，规模不断扩大，参会人数由最初的300余人，逐渐扩大至近2, 000人，形成了全国普胸外科最具影响力的行业和学术品牌会议。会议内容详实丰富、涵盖广泛，不仅包括专业学术，还涉及自律维权、医疗卫生体制改革、专科医师培训、学科建设等方面。

中国医师协会胸外科医师分会走过的十年，是我国普胸外科医师团结奋进的十年，也是我国传统胸外科向微创精准胸外科革命性过渡的十年，更是我国普胸外科逐步与国际接轨进而跨越走向引领地位的十年。

      中国医师协会胸外科医师分会

            第一、二届委员会会长

            第三届委员会前任会长


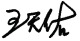


                        2018年3月


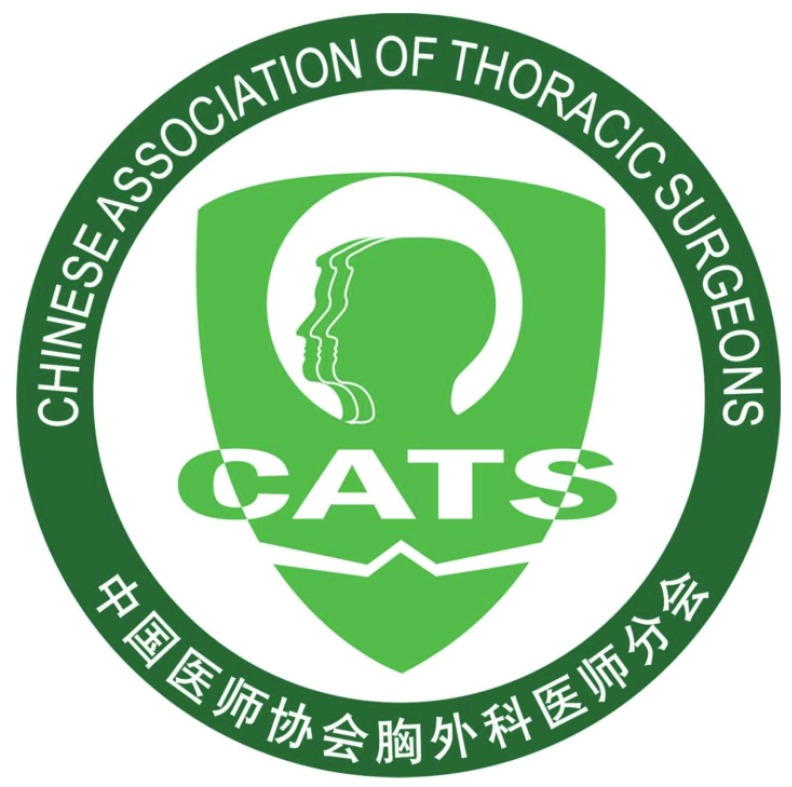


**中国医师协会胸外科医师分会简介**

中国医师协会胸外科医师分会(Chinese Association of Toracic Surgeons, CATS)是国家卫生部和民政部批准成立的中国胸外科医师全国性行业组织和社会团体，是中国注册胸外科医师的唯一行业性协会，是中国医师协会的二级机构。

中国医师协会胸外科医师分会由首都医科大学附属北京友谊医院王天佑教授负责筹备(包括王天佑教授、支修益教授、张志庸教授、甄文俊教授、张逊教授、高文教授、周允中教授、戎铁华教授、叶玉坤教授九人)，于2007年在北京成立，组成第一届委员会，王天佑教授担任第一届委员会会长。著名胸外科老前辈辛育龄教授、黄国俊教授为名誉会长，副会长为支修益(兼总干事)、张志庸、戎铁华、高文、叶玉坤、张逊、甄文俊、周允中教授。

2010年分会按照章程换届，成立第二届委员会。首都医科大学附属北京友谊医院王天佑教授连任分会第二届委员会会长，副会长为高文、戎铁华、王如文、叶玉坤、张临友、张逊、甄文俊、支修益教授(拼音排序)。

2014年分会再次换届，成立第三届委员会。天津胸科医院张逊教授担任分会第三届委员会会长，首都医科大学附属北京友谊医院王天佑教授为前任会长、中国医学科学院肿瘤医院赫捷院士为侯任会长。副会长为陈克能、费苛、傅剑华、高文、侯生才、李简、刘伦旭、毛伟敏、王俊、王如文、许林、张临友教授(拼音排序)。

中国医师协会胸外科医师分会的宗旨：发挥行业指导、服务、自律、协调、监督作用；团结和组织全国胸外科医师遵守国家宪法、法律、法规和政策；弘扬以德为本、救死扶伤人道主义的职业道德；维护医师的合法权益；努力提高医疗水平和服务质量；为全国人民的健康和社会主义中国肺癌杂志建设服务。

中国医师协会胸外科医师分会的主要任务：

(一)团结和组织广大医师，认真贯彻执行《中华人民共和国执业医师法》，通过实践，认真总结经验，向政府提供反馈意见。

(二)实行行业自律性管理，制定胸外科医师执业规范。协助卫生行政部门建立胸外科医师考核体系，审查、认证胸外科医师执业资格，监督检查执业情况。积极探索医师队伍管理的新模式、新方法，加强医师队伍的建设。

(三)依法维护胸外科医师在执业活动中享有的合法权益。努力营造和谐有序的医疗环境和医疗秩序，更好地为人民健康服务。使胸外科医师的劳动得到全社会的尊重。

(四)开展对胸外科医师的医学终身教育。

(五)积极开展医学科普宣传教育，推广医学科普知识，反对和批判封建迷信、伪科学。

(六)关心和帮助农村、基层的卫生工作，促进其预防、医疗水平不断提高。

(七)开展业务咨询服务，兴办为会员服务的机构。介绍推广医、药新技术、新成果，创办杂志刊物，促进医学科学技术的进步和发展。

(八)开展与国际及港澳台地区的医学交流与合作，学习借鉴先进的管理经验，更好地为广大医师服务。

(九)表彰和奖励在医疗、预防、保健工作中做出突出贡献的胸外科医师以及优秀的协会工作人员。

(十)调查并了解胸外科医师队伍的现状、要求和愿望。积极向中国医师协会和有关政府部门提出建设性意见，更好地调动和发挥广大胸外科医师的积极性。

(十一)努力争取和承办卫生行政部门和中国医师协会委托的有关工作以及与本会宗旨有关的事宜。

**中国医师协会胸外科医师分会历届年会大事记**

               **(2007-2016)**

2007年，北京年会，承办：首都医科大学附属北京友谊医院

中国医师协会胸外科医师分会成立，组建第一届委员会

2008年，江苏南京年会，承办：解放军南京八一医院

2009年，天津年会，承办：天津市胸科医院

2010年，广东东莞年会，承办：中山大学肿瘤医院

中国医师协会胸外科医师分会换届，组建第二届委员会

2011年，上海年会，承办：同济大学附属上海肺科医院

2012年，重庆年会，承办：第三军医大学大坪医院

2013年，四川成都年会，承办：四川大学华西医院

2014年，北京年会，承办：北京医师协会胸外科医师分会、

北京大学第一医院、北京大学肿瘤医院、首都医科大学附属北京朝阳医院

中国医师协会胸外科医师分会换届，组建第三届委员会

2015年，浙江杭州年会，承办：浙江省肿瘤医院

2016年，河南郑州年会，承办：河南省肿瘤医院

中国医师协会胸外科医师分会第一届青年委员会成立

